# Magnetic Resonance Imaging Manifestations of Pediatric Purulent Meningitis Based on Immune Clustering Algorithm

**DOI:** 10.1155/2022/9751620

**Published:** 2022-03-10

**Authors:** Dafei Wei, Pan He, Qian Guo, Yuanlu Huang, Hongxia Yan

**Affiliations:** Department of Pediatrics, The Second Affiliated Hospital of Nanhua University, Hengyang 421000, Hunan, China

## Abstract

The purpose of this study was to analyze the diagnostic value of magnetic resonance imaging (MRI) based on the immune clustering algorithm (ICA) in children with purulent meningitis. In this study, 235 children with suspected pediatric purulent meningitis (PPM) were routinely scanned, and the artificial immune algorithm (AIA) and ICA were applied to image processing. In order to quantitatively analyze the accuracy and precision of the processed image, precision rate was introduced as the evaluation of accuracy, and the True Positive Vis Fox, False Negative Vis Fo, and False Positive Vis Fo were selected as the evaluation indicators. After comparison, the accuracy, sensitivity, and specificity of ICA detection were higher than those of AIA and conventional plain scanning, and the differences were statistically obvious (*P* < 0.05). Comparison on image display effects showed that compared with AIA, the image processed by the ICA algorithm constructed in this study showed the highest definition and contrast and the best denoising effect and image quality, showing a statistically obvious difference (*P* < 0.05). All in all, the display effect of MRI images of pediatric purulent meningitis based on ICA was more accurate and clearer than that of the traditional image processing, and it can provide a more accurate auxiliary basis in the diagnosis of lesion details. It also showed a higher clinical value for the development of a diagnosis and treatment plan for complicated PPM.

## 1. Introduction

Purulent meningitis is an acute inflammatory response syndrome caused by the infection of the central nervous system by purulent bacteria or caused by the meninges [1, 2]. Severe cases lead to neurological sequelae, and more severe cases can cause death [3]. Purulent meningitis shows the clinical characteristics of acute onset, dangerous condition, and high mortality and disability rate [4]. A study [5] reported that about 26% of children with purulent meningitis survived with neurological sequelae. Related research [6] pointed out that magnetic resonance imaging (MRI) examination of the head is an important examination method for PPM, which plays an important role in clinical diagnosis, analysis, and treatment evaluation. MRI shows the following advantages for brain imaging [7]: studying the pathological conditions of the brain, formulating a radiotherapy plan, simulating surgery, observing, measuring the brain structure of the human body, and researching on the development of the human brain. Imaging the brain can accurately extract the patient's lesion information and digitally convert it to provide an important reference for clinical diagnosis [8]. Clear-edge information cannot be accurately obtained through MRI scanners. With the continuous development of artificial intelligence (AI) in recent years, neural network algorithms, ant colony algorithms (CAs), particle swarm optimization (PSO), genetic algorithms (GAs), immune algorithms (IAs), and other algorithms are combined with medical image processing, showing great value in medical examination and screening [9].

The artificial immune algorithm (AIA) is a bionic algorithm proposed by simulating the intelligent behavior of learning and memory of the biological immune system [10]. Its working mechanism is to search for the global optimal solution. When a similar problem reappears, the immune memory cell can quickly search for the optimal solution of the problem [11]. Clustering is to classify a data set according to similar features. The data information in the same category must have the greatest possible similarity under a certain feature, and the information in different categories must ensure as little consistency as possible [12]. With the general recognition and application of clustering technology, many related algorithms have been developed and improved by researchers in order to adapt to different scenarios [13]. Due to the complexity of such algorithms, it is difficult to accurately divide the existing clustering algorithms [14, 15]. In previous studies, it was found that AIA's global optimization performance is better, which is mainly reflected in its good self-adaptation and self-organization capabilities. However, its local search ability is not good, and super individuals are prone to appear in the early population of the algorithm, leading to premature convergence phenomena such as reduced population diversity [16], resulting in the application of some complex medical images, where the gray level difference is not obvious or image segmentation problems such as excessive overlap of grayscale ranges. The K-means algorithm is a common clustering algorithm with good local search capabilities [17], so AIA was combined with K-means clustering in this study to propose an MRI based on the immune clustering algorithm. The K-means clustering (KMC) algorithm is easy to implement and shows strong local search capabilities, but it is more sensitive to the selection of initial clustering centers. The AIA is combined with KMC to propose the ICA-based imaging method.

Therefore, 235 patients with suspected PPM were selected as the research objects and performed with a conventional MRI scan, an AIA-based MRI examination, and an ICA-based MRI examination in this study to comprehensively evaluate the diagnostic value of PPM through indicators such as display clarity and diagnostic accuracy.

## 2. Materials and Methods

### 2.1. Research Objects

In this study, 235 children with suspected PPM who were admitted to the hospital from January 2017 to December 2020 were selected as the research objects, including 128 males and 107 females. They were performed with a conventional MRI scan, an AIA-based MRI examination, and an ICA-based MRI examination. This study had been approved by the Ethics Committee of the hospital, and the family members of the children were aware of the study and signed the informed consent forms.

The inclusion criteria were defined as follows: patients with detailed and accurate clinical data; patients with complete imaging data; patients with no relevant treatment; children without contraindications to MRI.

The exclusion criteria were defined as follows: children who had congenital malformations; children with immune deficiency or fungal or cytomegalovirus infection; and patients who did not undergo head MRI within 48 hours of confirmed PPM.

### 2.2. Imaging Inspection

3.0 T superconducting magnetic resonance scanner was adopted in this study, which was equipped with an eight-channel head phased array coil. The patient was required to fast for at least 4 hours before the examination. The scanning parameters were given as follows: For plain scan (cross section), the time of repetition (TR) was 2430 ms, the time of echo (TE) was 25 ms, the layer thickness was 6 mm, and layer spacing was 1 mm; for cross section sequence, TR was 5524 ms, TE was 108 ms, layer thickness was 6 mm, and layer spacing was 1 mm; for cross section MRI fluid attenuated inversion recovery (FLAIR) sequence, the TR and TE were 8980 ms and 90 ms, respectively, and the layer thickness and spacing were 6 mm and 1 mm, respectively; for diffusion-weighted imaging (DWI) sequence, TR was 5110 ms, TE was 85 ms, the slice thickness was 6 mm, and the slice distance was 1 mm; and for MRI sequence, TR was 23 ms, TE was 3.6 ms, and the slice thickness was 1.6 mm.

All children were given a 5% chloral hydrate (1 mL/kg) enema 30 minutes before the examination, and the scan data was transferred to the processing workstation for processing.

### 2.3. MRI Image Algorithm Based on AIA

Step 1: the problem identification was realized with the following steps: The maximum between-class variance (Ostu) function was set as the objective function, and the problem to be optimized was set as the antigen, and then the solution to the problem could be derived. The set of solutions was defined as the antibody.
Step 2: the MRI medical image was checked. If it was a color image, it had to perform the grayscale processing, and then perform normalization processing on the processed grayscale image. If it was not a color image, it could be directly normalized. The normalization process made the grayscale range between 0 and 1.
Step 3: since the gray value of the image was 0–255, 2^8^ = 256, the gray value of the MRI brain image was encoded in an 8-bit binary manner.
Step 4: the following parameters were set: population size (popsize) = 20, maximum reproduction generation (maxgen) = 200, crossover probability (*Q*_*c*_) = 0.6, and mutation probability (*Q*_*m*_) = 0.06.
Step 5: it would randomly generate an initial population *B*_*1*_ with a size of *M*, and the affinity was adopted to measure the quality of the initial antibody. Here, the Ostu was used to compare each feasible solution in the population. Harmony was evaluated, and the antibody with the highest affinity was stored in immune memory cells. The affinity is calculated as follows:
  (1) $$\begin{array}{c} affinity=V_{0}*{\left(w_{0}-w\right)}^{2}+V_{1}*{\left(w_{1}-w\right)}^{2}. \end{array}$$
In equation (1), *V*_*0*_ was the proportion of the target pixel in the image; *w*_0_ referred to the average gray level of the target pixel; *V*_*1*_ represented the proportion of the background pixel in the image; *w* was the average gray level of the background pixel; and *V* referred to the total average gray, which is calculated as follows:
  (2) $$\begin{array}{c} V=v_{0}*w_{0}+v_{1}*w_{1}. \end{array}$$
Step 6: it should judge whether the end condition was met according to the degree of affinity. If the condition was met, the final optimization result would be output; otherwise, it would go back to step 5.
Step 7: the antibody concentration *D*_*i*_ was calculated with the following equation: *D*_*i*_ = *The affinity with antibody I is greater than the sum of the number of antibodies*
  (3) $$\begin{array}{c} \lambda /N. \end{array}$$
In equation (3), *λ* was the affinity constant, which met 0.9 ≤ *λ* ≤ 1, and *N* referred to the total number of antibodies.
The affinity between antibody *i* and antibody *j* can be calculated by the following equation:
  (4) $$\begin{array}{c} \left(A_{i}\right)=\frac{1}{1+m_{i}}, \end{array}$$
where *m*_*i*_ represents the binding strength among different antibodies, which can be calculated by Hamming distance (as follows):
  (5) $$\begin{array}{c} m_{i}=\sum_{i=1}^{L}\delta ,\left\{\begin{array}{c} \delta =1,ab_{i}\neq ag_{i}\\ \delta =0,ab_{i}=ag_{i} \end{array}.\right. \end{array}$$
Step 8: fitness′ was the result of the balanced evaluation of the affinity between the antibody and the antigen and the antibody self-concentration, and the antibody fitness′ was calculated using the following equation:
  (6) $$\begin{array}{c} fitness^{\prime }=affinity*\exp\left(k*D_{i}\right), \end{array}$$
fitness′ was essentially to correct the affinity between the antibody and the antigen. In equation (6), K referred to a negative number, and *k* = −0.8 was determined in this study.
Step 9: the initial antibody group B1 was sorted from large to small according to the value of aggregation fitness, and m individuals with high aggregation fitness were selected and copied according to the clone ratio. In this study, the number of candidate clones Mc was taken to 10, generating the cloned antibody group Ck.
Step 10: it can randomly generate an antibody group. The cloned antibody group Ck was undertaken as a vaccine, and the randomly generated antibody group to exchange genes at randomly selected positions according to the crossover probability to generate a crossover antibody group. According to the mutation probability, the position of an individual gene mutation in the antibody group after crossover was randomly determined, and then the gene value of the mutation point was reversed to generate the antibody group *D*_*k*_ after the mutation operation. In this study, *Q*_*c*_ *=* 0.8 and *Q*_*m*_ *=* 0.05.
Step 11: the individual affinity of each antibody in *D*_*k*_ was calculated, its size was sorted, and the antibody with the highest individual affinity was selected to update the memory cells.

When the algorithm was executed to the maximum number of iterations, the maximum affinity between the antigen and the antibody did not change for several consecutive generations, the algorithm stopped running.

### 2.4. MRI Image Algorithm Based on ICA

The following parameters were set: population size (popsize) = 30, maximum reproduction generation (maxgen) = 300, crossover probability (*Q*_*c*_) = 0.6, and mutation probability (*Q*_*m*_) = 0.06.

Step 1: the problem identification was realized with the following steps: The Euclidean distance *J* was taken as the objective function and the problem to be optimized was set as the antigen, and then the solution to the problem can be derived. The set of solutions was defined as the antibody.
Step 2: the MRI medical image was checked. If it was a color image, it had to perform the grayscale processing, and then perform normalization processing on the processed grayscale image. If it was not a color image, it could be directly normalized. The normalization process made the grayscale range between 0 and 1.
Step 3: since the gray value of the image was 0–255, 2^8^ = 256, the gray value of the MRI brain image was encoded in an 8-bit binary manner.
Step 4: the following parameters were set: popsize = 30, maxgen = 300, *Q*_*c*_ *=* 0.6, and *Q*_*m*_ *=* 0.06.
Step 5: it would randomly generate an initial population *B*_*1*_ with a size of *M*, and the affinity was adopted to measure the quality of the initial antibody. Here, each feasible solution in the population was selected to evaluate the affinity, the antibody with the highest affinity was saved in the immune memory cell, and the initial value of the antibody *J*_*1*_ was calculated as follows:
  (7) $$\begin{array}{c} affinity=\frac{1}{1+s_{ji}}. \end{array}$$
In the equation (7), *s*_*ji*_ was the Euclidean distance between the antibody and the antigen, which can be calculated by the following equation:
  (8) $$\begin{array}{c} s_{ji}=\sum_{j=1}^{K}\left(\sum_{i,j\in cj}||x_{i}^{\left(j\right)}-D_{j}||2\right). \end{array}$$
In equation (8), *x*_i_^(j)^ was the data object sample belonging to group *j*, *m*_*j*_ was the number of middle samples in group *j*, and *D*_*j*_ refers to the found cluster center.
As given in equation (8), the smaller the *s*_*ji*_, the greater the affinity.
Step 6: it should judge whether the end condition was met according to the degree of affinity. If the condition was met, the final optimization result would be output; otherwise, it would go back to step 5.
Step 7: the antibody concentration *D*_*i*_ was calculated with the following equation: *D*_*i*_ = The affinity with antibody I is greater than the sum of the number of antibodies
  (9) $$\begin{array}{c} \lambda /N. \end{array}$$
In equation (9), *λ* was the affinity constant, which met 0.9 ≤ *λ* ≤ 1, and *N* referred to the total number of antibodies.
The affinity between antibody *i* and antibody *j* can be calculated by the following equation:
  (10) $$\begin{array}{c} \left(B_{i}\right)=\frac{1}{1+s_{i}}. \end{array}$$
*s*_*i*_ represents the binding strength among different antibodies, which can be calculated by Euclidean distance:
  (11) $$\begin{array}{c} s_{ji}=\sum_{j=1}^{K}\left(\sum_{i,j\in c_{j}}||x_{i}^{\left(j\right)}-x_{j}^{\left(j\right)}||2\right). \end{array}$$
Step 8: fitness′ was the result of the balanced evaluation of the affinity between the antibody and the antigen and the antibody self-concentration, and the antibody fitness′ was calculated using equation:
  (12) $$\begin{array}{c} fitness^{\prime }=affinity*\exp\left(k*D_{i}\right). \end{array}$$
Fitness′ was essentially to correct the affinity between the antibody and the antigen. In equation (12), *k* was a negative number, and *k* = −0.8 was determined in this study.
Step 9: the initial antibody group *B*_*1*_ was sorted from large to small according to the value of aggregation fitness, and *m* individuals with high aggregation fitness were selected and copied according to the clone ratio. In this study, the number of candidate clones *M*_*c*_ was taken as 10, generating the cloned antibody group *C*_*k*_.
Step 10: it can randomly generate an antibody group. The cloned antibody group *C*_*k*_ was undertaken as a vaccine and the randomly generated antibody group to exchange genes at randomly selected positions according to the crossover probability to generate a crossover antibody group. According to the mutation probability, the position of individual gene mutation in the antibody group after crossover was randomly determined, and then the gene value of the mutation point was reversed to generate the antibody group *D*_*k*_ after the mutation operation.
Step 11: the individual affinity of each antibody in *D*_*k*_ was calculated, its size was sorted, and the antibody with the highest individual affinity was selected to update the memory cells. Then, the objective function value *J*^*∗*^ of the antibody was calculated.

When the algorithm was executed to the maximum number of iterations or the difference between the initial target value *J*_*1*_ of the antibody in the memory cell and the target function value *J*^*∗*^ of the antibody after cross-mutation met the set threshold, the algorithm stopped and the antibody was decoded to get the best cluster center.

### 2.5. Statistical Analysis

The data in this study was processed by SPSS20.0 version statistical software. The measurement data was expressed in the form of mean ± standard deviation ($\bar{x}$ ± *s*), and the count data was expressed as percentage (%). One-way analysis of variance was used for pairwise comparison. The difference was statistically significant at *P* < 0.05.

## 3. Results

### 3.1. Analysis on Algorithm Performance

**Figure 1:**
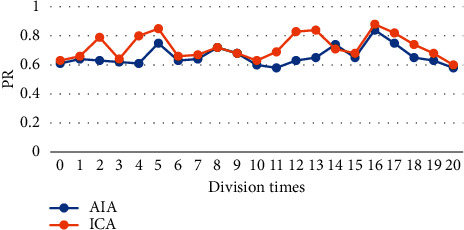
Changes of the PR of the three algorithms with the number of divisions.

**Figure 2:**
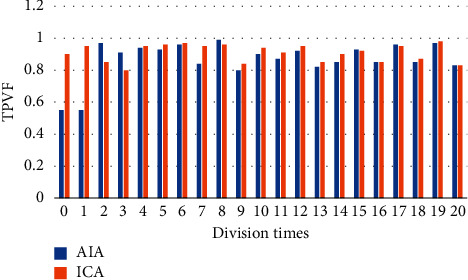
Changes of the TPVF of the three algorithms with the number of divisions.

**Figure 3:**
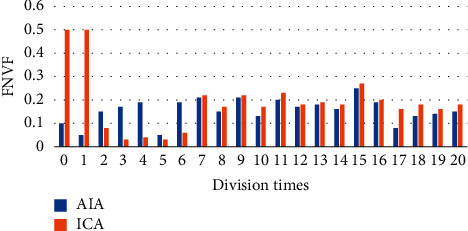
Changes of the FNVF of the three algorithms with the number of divisions.

**Figure 4:**
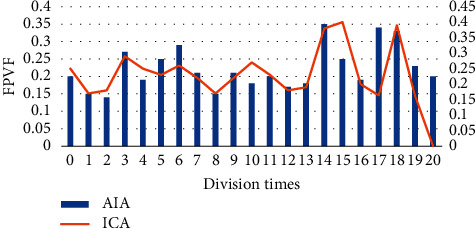
Changes of FPVF of the three algorithms as the number of divisions.

In order to quantitatively analyze the accuracy and precision of the processed image, precision rate (PR) was introduced as the evaluation of accuracy, and the True Positive Vis Fox (TPVF) and False Negative Vis Fo (FNVF), and False Positive Vis Fo (FPVF) were selected as the evaluation indicators [18, 19]. The AIA and ICA algorithms were used to perform 20 simulation experiments on the flat-scan image, respectively, and the same objective function was selected.  Figure 1 shows the changes of the PR of the three algorithms with the number of divisions, Figure 2 shows the changes of the TPVF of the three algorithms with the number of divisions, Figure 3 shows the changes of the FNVF of the three algorithms with the number of divisions, and Figure 4 illustrates the FPVF of the three algorithms as the number of divisions changes.

**Figure 5:**
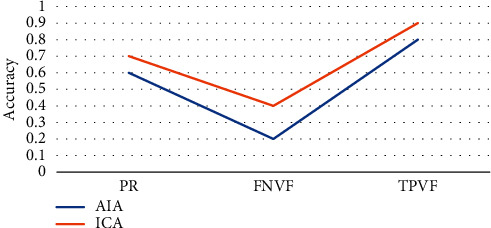
Comparison on the average values of accuracy and precision.

According to the above simulation results, the accuracy and precision were averaged. As shown in Figure 5, the PR and TPVF of the ICA algorithm were obviously better than those of the AIA algorithm.

### 3.2. Comparison on Image Display Effect

**Figure 6:**
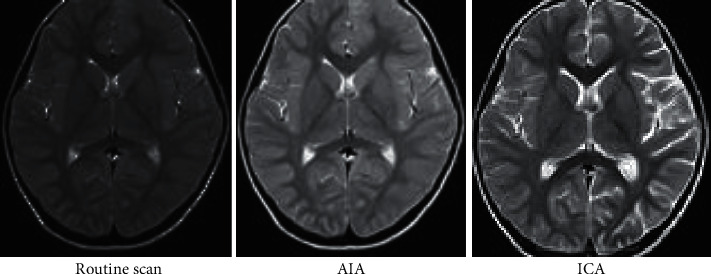
Comparison on the enhancement effect of algorithms on MRI images.

As shown in Figure 6, the image processed by the ICA algorithm shows the highest contrast and the best denoising effect.

### 3.3. Comparison on Detection Rate

**Figure 7:**
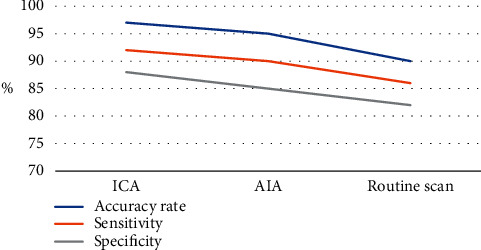
Comparison on detection effects of various algorithms.

As shown in Figure 7, the accuracy, sensitivity, and specificity of the MRI images processed by the conventional plain scan, AIA, and ICA were compared. The accuracy, sensitivity, and specificity of ICA detection were slightly higher than those of AIA and conventional plain scan, and the differences between the two were not statistically remarkable (*P* > 0.05).

### 3.4. Comparison on Display Clarity

**Figure 8:**
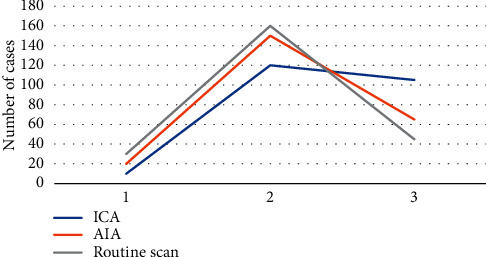
Comparison on display clarity. *Note.* 1, 2, and 3 in the figure referred to the number of cases with unclear display, clear display, and very clear display, respectively.

Figure 8 illustrates the comparison results for conventional plain scan, AIA, and ICA on the display clarity of the cranial region. Compared with AIA, the number of cases with unclear display of the cranial area of ICA and clear display was statistically obvious (*P* < 0.05). The number of cases with conventional plain scan showing unclear brain area was statistically different from that of AIA (*P* < 0.05), and the number of cases of conventional plain scan showing clearer tumor neck area and the number of cases showing very clearly showed no difference compared with ICA (*P* > 0.05).

## 4. Discussion

Purulent meningitis is a common pediatric intracranial nervous system infectious disease, which is clinically called bacterial meningitis. Its fatality rate and disability rate are very high; hence, it is extremely important for patients to be treated on time as the patients are under tremendous pressure. At present, the main clinical examination method for purulent meningitis is to use MRI examination [20]. In order to improve the clarity of imaging, the conventional MRI scan, AIA-based MRI, and ICA-based MRI were adopted in this study, and their performance, imaging effect, detection rate, and display clarity were compared. It was found that with the same function, the PR, TPVF, and FNVF suggested that ICA showed an advantage over AIA, and the differences were statistically dramatic (*P* < 0.05). In terms of the actual display effect of the image, whether it was the AIA algorithm or the ICA algorithm, the denoising of the original image showed obvious effects. the ICA algorithm constructed in this study showed the best denoising effect on the image, and the processed image was even more clear, with greatly reduced artifacts and improved quality [21].

The research results of this study proved that the accuracy, sensitivity, and specificity of ICA were slightly higher than those of AIA, which is ahead of the original detection method; however, the difference between the two was not statistically visible (*P* > 0.05). It indicated that whether it was based on the ICA algorithm or the AIA algorithm, the detection results were similar, and there was no obvious difference between the superior and the inferior. However, compared with conventional inspections, ICA showed higher specificity for PPM, the gap was larger, and the difference was obvious statistically (*P* < 0.05). Such results are similar to the results of Fukuda et al. (2020) [22], indicating that the examination effect based on the ICA algorithm can provide a more accurate auxiliary basis for the diagnosis of lesion details due to the original method. From the perspective of the display clarity of the three algorithms, the ICA display was not clear, and the number of clear display cases was statistically greater compared with the conventional plain scan (*P* < 0.05), and the number of unclear cases of the conventional plain scan on the brain area was statistically different from that of AIA (*P* < 0.05). Such results prove that the MRI imaging effect based on the ICA algorithm is far superior to the conventional plain scan.

## 5. Conclusion

Children with purulent meningitis who have conventional blood tests and cerebrospinal fluid tests can be found to have more prominent abnormal features, and multiple pathologies lead to multiple features in imaging. As the most common diagnostic method of imaging, MRI plays a very important role in the diagnosis and treatment evaluation of craniocerebral nervous system diseases. In this study, the AIA and ICA were introduced through the biological immune system and the KMC algorithm, and they were applied to image graphics processing. PR was introduced as the evaluation of accuracy to quantitatively analyze the accuracy and precision of the processed image. The TPVF, FNVF, and FPVF were undertaken as evaluation indicators to analyze the detection accuracy, sensitivity, and specificity of the algorithms. It was found that the accuracy, sensitivity, and specificity of the ICA were slightly higher than those of the AIA and conventional plain scan. The comparison revealed that the ICA algorithm constructed in this study showed the highest image definition and contrast and the best denoising effect and image quality. However, the depth of the study was slightly insufficient due to the small sample size. Later, more children's data would be collected for research to improve the reliability of the research results. All in all, this study provided a better algorithm for brain MRI image analysis, which greatly improved the imaging quality and was worthy of promotion in clinical applications.

## Data Availability

The data used to support the findings of this study are available from the corresponding author upon request.
